# Vitamin D Deficiency and Its Association With Cardiovascular Diseases Among Patients Attending a Private Tertiary Sector Cardiovascular Heart Clinic in Nairobi

**DOI:** 10.7759/cureus.43012

**Published:** 2023-08-06

**Authors:** Yubrine M Gachemba, Zahid Khan, Elijan Njau, Martin Wanyoike

**Affiliations:** 1 Cardiology, The Nairobi Hospital, Nairobi, KEN; 2 Acute Medicine, Mid and South Essex NHS Foundation Trust, Southend-on-Sea, GBR; 3 Cardiology, Barts Heart Centre, London, GBR; 4 Cardiology and General Medicine, Barking, Havering and Redbridge University Hospitals NHS Trust, London, GBR; 5 Cardiology, Royal Free Hospital, London, GBR

**Keywords:** risk factors of cardiovascular diseases, vitamin d deficiency, awareness of cardiovascular diseases, cardiometabolic diseases, kenyatta national hospital, high-density lipoprotein, framingham cardiovascular risk score, retrospective cross-sectional study, cardiovascular disease and vitamin d deficiency, cardiovascular disease prevention

## Abstract

Background: Vitamin D deficiency is a common condition that affects approximately 30-50% of the worldwide population. Vitamin D deficiency is associated with an increased risk of cardiometabolic diseases and is considered a cardiovascular risk factor globally.

Methods: This is a retrospective cross-sectional study that aimed to identify the prevalence of vitamin D deficiency and its associations with the cardiovascular disease (CVD) risk profile of patients presenting for cardiac evaluation at Primecare Heart Clinic, a private heart clinic in Nairobi, Kenya, between January 1, 2020 and January 31, 2022.

Results: Females with vitamin D deficiency composed 58.87% of the study participants. The average 10-year Framingham CVD risk level of the vitamin D-deficient participants was 7.09%. Participants with vitamin D deficiency that were older and had low serum high-density lipoprotein C (HDL-C) levels and high systolic blood pressure (BP) had a higher risk of CVDs. Male participants were at five times higher risk of CVDs. Vitamin D-deficient patients who were older and had a low HDL cholesterol level and high systolic BP are at a high risk of CVDs. The two-way analysis of variance (ANOVA) test value was 345.6992, and the p-value was 0.002884.

Conclusion: Our study demonstrated that a low level of vitamin D was associated with a higher Framingham cardiovascular risk score and cardiovascular risk in patients. Nonetheless, this is a retrospective study, which is a major limitation of this study.

## Introduction

Vitamin D deficiency is a common condition affecting about 30-50% of the worldwide population [1,2]. Vitamin D deficiency has emerged as a novel cardiovascular disease (CVD) risk factor besides its role in calcium homeostasis [2]. Despite the available evidence, intervention trials involving vitamin D supplementation in patients with CVD risk remain controversial. The causal association between vitamin D deficiency and CVDs is supported by biological plausibility, the finding of a dose-response between 25-hydroxyvitamin D (25(OH)D) deficiency and CVD risk, and the demonstration of a temporal association between vitamin D and CVDs [3]. Correcting vitamin D deficiency with vitamin D supplements or lifestyle measures can result in CVD risk reduction [4]. A study on diabetic patients showed that vitamin D deficiency was associated with higher Framingham risk scores and hemoglobin A1C (HbA1C) [5]. The primary role of vitamin D is increasing intestinal calcium absorption for bone mineralization. 1,25-dihydroxy vitamin D (1,25(OH)2D), which is the active form of vitamin D, binds to the vitamin D receptors (VDRs) by acting as a steroid hormone that is present in many body cells, such as cardiomyocytes, vascular smooth muscle, and endothelial cells [6]. Recently, there has been a growing interest in the association of vitamin D with CVD risk, although the exact mechanism for this is unclear. The negative regulation of renin to lower blood pressure (BP), improve vascular compliance, decrease parathyroid hormone levels, and improve glycaemic control are a few proposed mechanisms [6].

The earliest evidence linking vitamin D deficiency to a higher risk of death from ischemic heart disease (IHD) comes from cross-sectional studies in 1980s and 1990s [7-10]. The first evidence came from a study by Fleck that recognized higher mortality rates in people with increasing distance from the equator [7]. A study in the United Kingdom found an inverse relationship between IHD and the number of hours of sunlight exposure [8]. Another study demonstrated a higher incidence and mortality rate from IHD in winter months when sunlight was low, showing a seasonal variation [9]. Rostand et al. noted a higher BP in individuals further away from the equator, suggesting that cutaneously synthesized vitamin D might have a regulatory role in BP [10].

Adamczak et al. suggested that vitamin D supplementation can promote a reduction in left ventricular hypertrophy (LVH), BP, and inflammatory cytokines [4]. However, vitamin D and calcium supplementation did not reduce cardiovascular events in a Women’s Health Initiative study [11].

Karuppusami et al. suggested that an assessment of CVD risk across a broad range of baseline 25(OH)D levels is necessary for clinical practice, and thus the motivation for additional data mainly from the African setup could identify surrogate endpoints or subgroups most likely to benefit from vitamin D supplementation [11]. Vitamin D deficiency is now considered a novel risk factor for CVDs [4,5]. Mirhosseini et al. reported that vitamin D exerts beneficial cardiovascular effects by reducing the renin-angiotensin-aldosterone system activation, leading to lower BP and anti-proliferative, anti-hypertrophic, anti-thrombotic, and anti-fibrotic effects [12]. In addition, vitamin D preserves endothelial function by inhibiting the proliferation of vascular smooth muscle cells through calcium influx [12]. A randomized controlled trial (RCT) demonstrated the protective effects of vitamin D in older patients from heart failure, but it also found that vitamin D does not protect against myocardial infarction (MI) or cerebrovascular accident (CVA) [13]. A study showed that repletion of vitamin D was associated with improved CVD outcomes [14]. Vitamin D deficiency is considered to be a global public health problem mainly affecting countries with a lack of sun, which is associated with an increased risk of cardiometabolic diseases [15]. Parker et al. performed a study to identify the prevalence of vitamin D deficiency and its associations with CVDs given the high prevalence of vitamin D deficiency across the globe as observed with the COVID-19 pandemic [16].

This retrospective descriptive cross-sectional study aimed to assess vitamin D deficiency and the CVD risk factor profile in Kenya. The primary objective of this study was to establish the CVD risk profile of patients with vitamin D deficiency presenting for cardiac evaluation at Primecare Heart Clinic in Nairobi, Kenya, between January 1, 2020 and January 31, 2022.

## Materials and methods

Ethical consideration

This study was approved by the Nairobi Hospital Ethics Committee and Kenya National Commission for Science Technology and Innovation (NACOSTI). Participants' confidentiality was strictly maintained, and the data were securely stored. The ethics committee approval number is TNH-ERC/DMSR/RP/030/22.

Methodology

A retrospective descriptive cross-sectional study was carried out in a private tertiary heart clinic housed by the Nairobi Hospital, Kenya. With a study frame of 6,400 patients, a sample of 124 patients with documented vitamin D deficiency had their 10-year Framingham CVD risk calculated. The Nairobi Hospital is a 750-bed large private referral hospital situated in the capital city of Kenya, Nairobi, that also serves as the teaching hospital for the Nairobi Hospital College of Health Sciences. There are about 12,000 patients on follow-up at the heart clinic, which sees approximately 250 patients a month. The clinic runs every weekday and Saturday from 8 am to 6 pm with approximately 20 to 40 patients seen per day, of which more than 70% have underlying CVDs. The study included patients who presented at the Primecare Heart Clinic for routine follow-up from January 1, 2020 to January 31, 2022. All patients with documented vitamin D deficiency in the stipulated period were included in the study. A sample size of 135 patients with documented vitamin D deficiency was eligible for the study enrollment following the application of inclusion and exclusion criteria. Eleven patients did not have serum lipid profile checked and hence were excluded from the study as it was not possible to calculate their CVD risk score due to the lack of the above necessary parameters. The remaining 124 patients had their 10-year Framingham CVD risk calculated. The sampling frame was generated from the daily appointment register, which is stored electronically in the electronic medical records (EMRs).

Inclusion Criteria

The inclusion criteria were patients attending Primecare Heart Clinic from January 1, 2020 to January 31, 2022, with documented vitamin D deficiency and aged over 30 years with or without additional risk factors.

Exclusion Criteria

The exclusion criteria were pregnant patients, aged >79 years, patients with CVDs and hyperparathyroidism, patients taking vitamin D supplementation before the clinic visit, diabetic patients, patients with a previous history of coronary artery disease, and patients with intermittent claudication or documented prior history of peripheral artery disease (PAD).

Primary Outcomes

The primary outcomes included the presence of any vitamin D deficiency.

Secondary Outcomes

The secondary outcome is the association of vitamin D deficiency to CVD risk factors.

Data management and analysis methods

Data from the EMRs were extracted for all adult patients who visited the clinic during the study period. The 10-year CVD risk was estimated using the Framingham risk score. Patients' EMRs were accessed, and data on patients' demographics, CVD risk, and latest vitamin D results were collected. Data from the patient records were entered into a study spreadsheet with a unique code. The final data analysis was performed using IBM SPSS Statistics for Windows, Version 23 (released 2015; IBM Corp., Armonk, New York, United States), and the analysis was undertaken to test the association between vitamin D deficiency and CVD risk of the patients.

The descriptive characteristics of the study population, such as age, gender, smoking status, medication for hypertension, and lipid profile, were summarized into percentages for categorical data and means or medians for continuous variables. The presence of vitamin D deficiency and CVD risk profile was analyzed and presented as proportions with 95% confidence intervals. We performed chi-square and analysis of variance (ANOVA) tests to assess the association between vitamin D deficiency and various patient variables.

## Results

Research findings and discussions

Demographics

**Figure 1:**
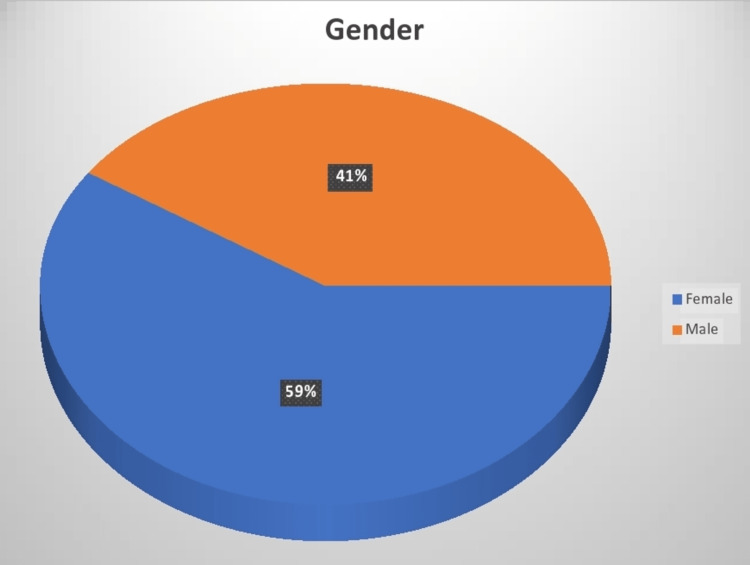
Vitamin D deficiency and gender

This study sought to determine the 10-year Framingham CVD risk profile of the vitamin D-deficient patients over the duration between January 1, 2020 and January 31, 2022 and describe the CVD risk characteristics and their association with vitamin D deficiency. The parameters used by the Framingham risk score profile included age, gender, use of antihypertensive therapy, smoking status, serum total cholesterol levels, and serum HDL levels and their contribution to the 10-year risk of CVD. Figure 1 shows that most participants who were vitamin D deficient were female 73 (58.87%), while their male counterparts accounted for the remaining 51 (41.13%).

**Figure 2:**
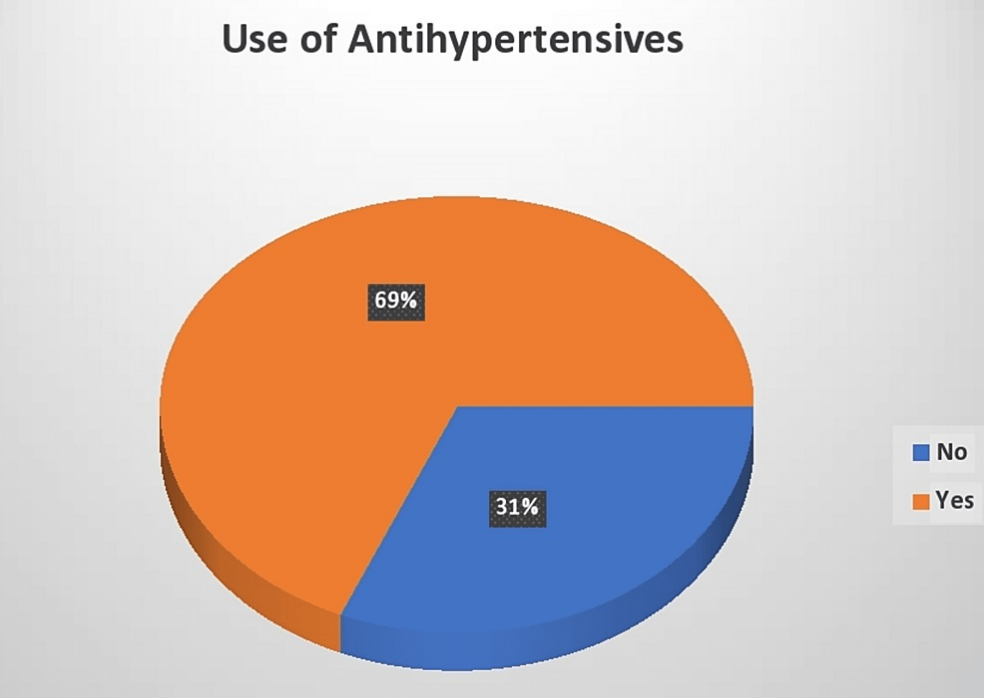
Vitamin D deficiency and the use of antihypertensive medications

The study found that 85 (68.55%) vitamin D-deficient patients were on antihypertensive therapy, whereas 39 (31.45%) patients were not on any antihypertensive therapy (Figure 2).

**Figure 3:**
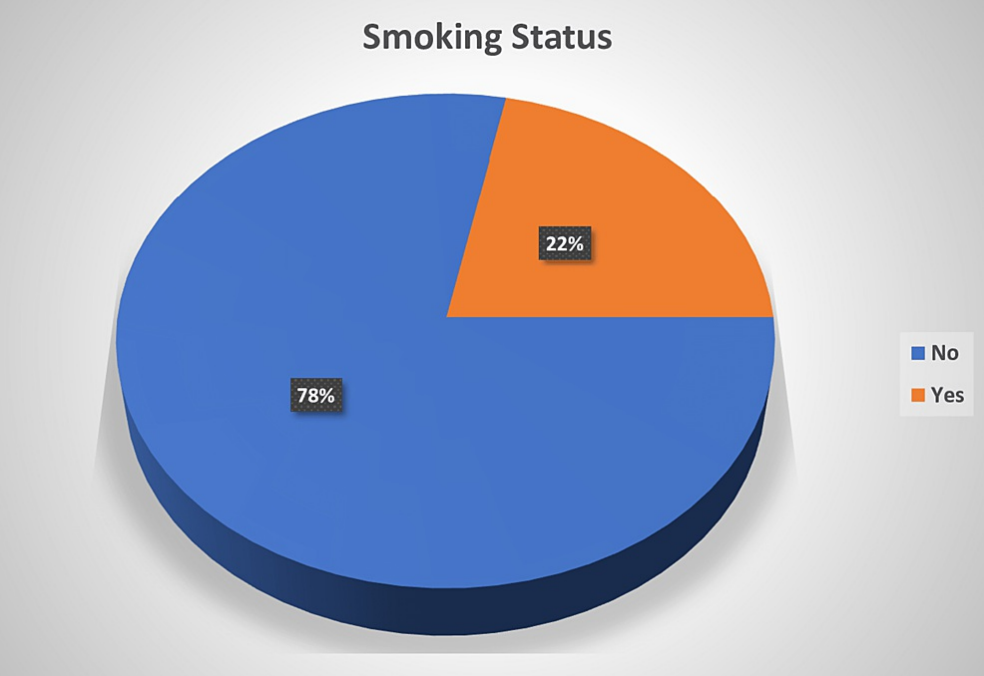
Vitamin D deficiency and smoking status

From vitamin D-deficient patients, 27 (21.77%) patients were current smokers, while the remaining 97 (78.23%) did not smoke, as shown in Figure 3.

**Figure 4:**
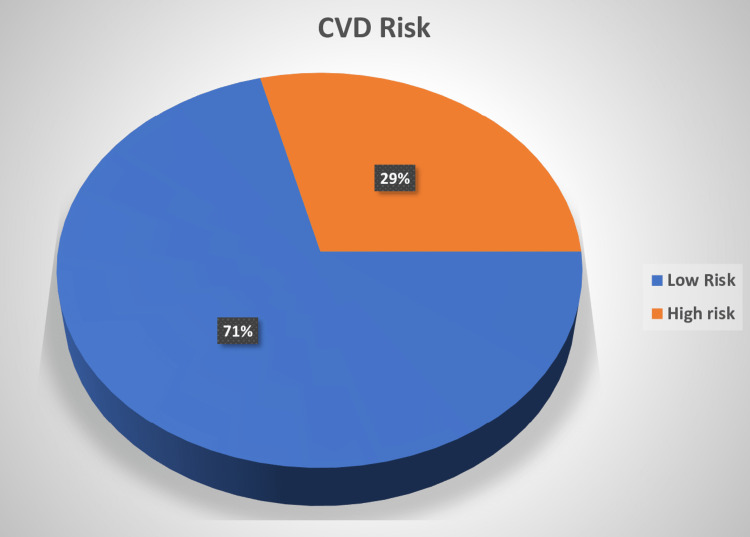
Vitamin D deficiency and cardiovascular risk

Thirty-six (29.03%) of the patients who were vitamin D deficient were found to have a high risk of CVDs compared to the remaining 88 (70.97%) who has low or moderate risk of CVDs (Figure 4).

Descriptive Statistics of Vitamin D-Deficient Participants

The descriptive statistics of the vitamin D-deficient participants targeted for this study are outlined in Tables 1-5. We found that the mean total cholesterol among the study participants was 4.93 mmol/L. A substantial proportion of 31.85% (43) of the participants was found to have a high total cholesterol of 5.2 mmol/L. The mean HDL cholesterol level for the participants was 1.197 mmol/. The study however noted that 25.93% of the participants had an HDL cholesterol level of less than 1, which is a risk factor for CVDs. The mean systolic BP level of the participants was 146.1 mmHg, indicating a significant high BP among the participants. The average age of the participants who were vitamin D deficient was 50.35 years with a variability of 12.88 years, with the youngest participant being 27 years old and the oldest being 79 years old.

**Table 1:** Descriptive characteristics HDL: high-density lipoprotein; BP: blood pressure; CVD: cardiovascular disease

Statistics						
		Total cholesterol (mmol/L)	HDL cholesterol (mmol/L)	Systolic BP (mmHg)	Age (years)	Framingham 10-year CVD risk
N	Valid	124	124	124	124	124
	Missing	0	0	0	0	0
Mean		4.9323	1.1973	146.1129	50.35	0.070919
Median		4.7000	1.2000	148.0000	48.00	0.029500
Mode		4.00	1.20^a^	123.00^a^	40	0.0010
Standard deviation		0.99082	0.33449	19.45903	12.875	0.0861154
Range		5.10	1.89	80.00	52	0.4150
Minimum		4.00	0.10	110.00	27	0.0010
Maximum		9.10	1.99	190.00	79	0.4160
a. Multiple modes exist. The smallest value is shown						

The average 10-year Framingham CVD risk level of the vitamin D deficient participants was found to be 7.09% over 10 years. Of these, 4.44% were found to have a higher risk at more than 20%. The study sought to further establish the factors that contributed to a higher risk of CVD amongst patients who were vitamin D deficient. Preliminary analysis showed that sex, use of antihypertensive therapy, and current smoking status combined with a deficiency of Vitamin D exacerbated the risk of CVD. Male participants were found to be 5 times more at risk of CVD compared to their female counterparts.

Table 2 shows that 86.11% (31) of the vitamin D deficient participants who were at high risk of CVD were male, accounting for the largest proportion amongst the two genders.

**Table 2:** Risk of CVDs by sex CVD: cardiovascular disease

			CVD risk		Total
			Low risk	High risk	
Sex	Female	Count	68	5	73
		% of total	54.8%	4.0%	58.9%
	Male	Count	20	31	51
		% of total	16.1%	25.0%	41.1%
Total		Count	88	36	124
		% of total	71.0%	29.0%	100.0%

The female participants with vitamin D deficiency were 3.5 times less likely to be at higher risk of CVDs compared to the male participants. The X^2^ value was 42.3907 for female with a p-value of <0.00001. Furthermore, we found that 68.50% (85) of the vitamin D-deficient participants were on antihypertensive therapy. Of these, 36.47% (31) were at a high risk of CVDs, as shown in Table 3.

**Table 3:** Risk of CVDs of those under antihypertensive therapy

			CVD risk		Total
			Low risk	High risk	
Antihypertensive therapy	No	Count	34	5	39
		% of total	27.4%	4.0%	31.5%
	Yes	Count	54	31	85
		% of total	43.5%	25.0%	68.5%
Total		Count	88	36	124
		% of total	71.0%	29.0%	100.0%

Smoking was another factor that was noted to contribute to the participants being at a higher risk of CVDs. Table 4 shows that 44.40% (12) of the vitamin D-deficient participants who smoked were at a higher risk of CVDs. Those who did not smoke were also at a high risk of CVDs, accounting for 24.74% (24) in that category. The X^2^ for smokers versus non-smokers was 3.9792, and the p-value was 0.046064.

**Table 4:** Risk of CVD by the smoking status

Smoking status			CVD risk		Total
			Low risk	High risk	
Smoker	No	Count	73	24	97
		% of total	58.9%	19.4%	78.2%
	Yes	Count	15	12	27
		% of total	12.1%	9.7%	21.8%
Total		Count	88	36	124
		% of total	71.0%	29.0%	100.0%

Thirty-six participants are at a high risk of CVDs, and 88 are at a low risk of CVDs. The mean age for patients who are at high risk of CVDs was 61.36 years, while those at a low risk were 45.85 years. The average HDL cholesterol level of vitamin D-deficient patients who were at a high risk of CVDs was 1.05 mmol/L, and that of low-risk patients was 1.2574 mmol/L. The systolic BP level of the patients with vitamin D deficiency who were at a high risk of CVD was 153.56 mmHg, and that of the patients with a low risk was 143.07 mmHg. Vitamin D-deficient patients who were older and had a low HDL cholesterol level and high systolic BP are at a high risk of CVDs (Table 5). The two-way ANOVA test value was 345.6992, degree of freedom (df) (df1 2, df2 2), and the p-value was 0.002884.

**Table 5:** Group statistics for CVD risks CVD: cardiovascular disease; BP: blood pressure; HDL: high-density lipoprotein

Group statistics					
CVD risk		N	Mean	Standard deviation	Standard error mean
Age	Low risk	88	45.85	10.311	1.099
	High risk	36	61.36	11.948	1.991
HDL cholesterol	Low risk	88	1.2574	0.33722	0.03595
	High risk	36	1.0503	0.28141	0.04690
Systolic BP	Low risk	88	143.0682	18.74622	1.99835
	High risk	36	153.5556	19.41346	3.23558

## Discussion

Our findings suggest a positive association of vitamin D deficiency with CVDs, and vitamin D deficiency was associated with increased CVD risk. Older patients, female gender, pre-existing hypertension, and abnormal lipid profile characterized by elevated total cholesterol and low HDL-C patients were more likely in vitamin D-deficient individuals. Soh et al. demonstrated that vitamin D deficiency increases the risk of developing incident hypertension or cardiovascular mortality in individuals with pre-existing CVDs [15,16].

Previous studies focused on vitamin D supplementation as a potential antihypertensive agent [17,18]. An RCT conducted in elderly German women demonstrated 9% BP reduction by taking a modest amount of vitamin D (400 IU) plus calcium given for eight weeks [19]. Schwab et al. randomized 18 subjects with stage I hypertension to ultraviolet A (UVA) exposure to the skin and ultraviolet B (UVB) exposure to the skin and demonstrated a significant reduction in both systolic and diastolic BP after six weeks of UVB therapy. The UVA therapy did not produce vitamin D, whereas UVB produced vitamin D, suggesting that the cutaneously produced vitamin D can lower BP.

Contrary to our study’s findings, the Women’s Health Initiative study conducted in the United States did not demonstrate any significant decrease in systolic or diastolic BP in women randomized to calcium and vitamin D (400 IU) at the end of a seven-year follow-up [20]. Thus, vitamin D deficiency has not consistently been demonstrated to have a positive effect on regulating BP. As already mentioned, vitamin D insufficiency is quite common across the globe, with available evidence from epidemiologic studies suggesting a strong association between diabetes risk, CVD risk, metabolic syndrome, and vitamin D deficiency. As noted from our findings, smoking and vitamin D deficiency cumulatively increase the predisposition to increased risk of CVDs. CVD burden can be attributed to certain associated behaviors, such as a sedentary lifestyle, unhealthy dietary patterns, smoking, and excessive alcohol consumption. Growing evidence supports significant cause-and-effect relationships between most of these factors with CVDs [21,22].

Female patients were more likely to be vitamin D-deficient in this clinical audit. These findings are consistent with the previously available data [23]. The possible explanations for these deficiencies are the lack of exposure to sunlight due to covering of the body seen in women of African cultural background and possibly hormonal changes noted in postmenopausal women. This study demonstrated an association between high cholesterol levels and vitamin D deficiency among CVD patients. These findings contrast with those from previous data [24], which did not demonstrate any such association between low vitamin D and high cholesterol levels in obese patients.

The likely explanation for this is that low levels of parathyroid hormones increase the levels of intracellular calcium in adipocytes, which leads to obesity due to lipogenesis [25]. We should be mindful that the Framingham 10-year risk calculator does not include the body mass index (BMI) as a parameter in the risk score calculator. As noted from the findings, dyslipidemia is often associated with vitamin D deficiency. This finding is likely explained by an increase in hepatic cholesterol production secondary to decreased transcriptional activity of the VDR [26].

Therefore, the study’s findings suggest that the risk factors of CVD among vitamin D-deficient patients need monitoring to prevent CVD morbidity and mortality. It may be useful to develop regional and national health promotion measures to increase awareness about vitamin D deficiency among the general population and especially CVD patients [27,28,29]. Future studies and trials will be able to guide how to manage vitamin D status in clinical practice. Likely, increasing vitamin D status with a daily supplement containing at least 1000 IU may be sufficient for the general population, particularly in areas with increased latitude from the equator or during the winter months. Whether this is a cost-effective practice is a subject that needs further interrogation and clinical evidence as there is still a scarcity of data concerning this practice in cardiovascular medical practice.

Strengths and limitations of this study

It is the first retrospective study of its kind conducted in Kenya to explore the association of CVDs and their risk factors associated with vitamin D deficiency. There were also several limitations to our study, including its retrospective design and several patients not meeting the specific inclusion criteria for inclusion based on baseline characteristics. Thus, we could not determine the exact causal inference or temporality of association between variables. The study sample size was also small, likely attributable to the change in health-seeking behaviors among patients during the peak of the COVID-19 pandemic and the short study time frame, which could explain the significant results. This likely reduced the generalizability of the study findings. In addition, we included only patients with vitamin D deficiency, and there was no control group, which made the interpretation difficult.

Recommendations

Based on the findings, it appears that the role of vitamin D for CVD prevention in the general population still elicits many unanswered questions and will likely fuel more research and investigations into this debate. The true role and benefit of vitamin D repletion on CVD outcomes need to be established and is likely to keep our minds engaged concerning the role of vitamin D in cardiovascular practice for the near future.

## Conclusions

Vitamin D deficiency has been implicated in the pathogenesis and complications of CVDs. The mechanism of how serum vitamin D levels could be an effective target for CVD prevention is still an extensive biologic plausibility theory. Many observational studies and large randomized placebo-controlled trials in the general population have refuted this theory. Thus, based on available data on vitamin D, supplementation for CVD prevention is not recommended for the public.
